# Computational design of biopolymer aerogels and predictive modelling of their nanostructure and mechanical behaviour

**DOI:** 10.1038/s41598-021-89634-1

**Published:** 2021-05-13

**Authors:** Rajesh Chandrasekaran, Markus Hillgärtner, Kathirvel Ganesan, Barbara Milow, Mikhail Itskov, Ameya Rege

**Affiliations:** 1 Department of Continuum Mechanics, RWTH Aachen University, Eilfschornsteinstr. 18, 52062 Aachen, Germany; 2 Department of Aerogels and Aerogel Composites, Institute of Materials Research, German Aerospace Center, Linder Höhe, 51147 Cologne, Germany

**Keywords:** Nanoscale materials, Mechanical properties, Computational methods, Gels and hydrogels

## Abstract

To address the challenge of reconstructing or designing the three-dimensional microstructure of nanoporous materials, we develop a computational approach by combining the random closed packing of polydisperse spheres together with the Laguerre–Voronoi tessellation. Open-porous cellular network structures that adhere to the real pore-size distributions of the nanoporous materials are generated. As an example, κ-carrageenan aerogels are considered. The mechanical structure–property relationships are further explored by means of finite elements. Here we show that one can predict the macroscopic stress–strain curve of the bulk porous material if only the pore-size distributions, solid fractions, and Young’s modulus of the pore-wall fibres are known a priori. The objective of such reconstruction and predictive modelling is to reverse engineer the parameters of their synthesis process for tailored applications. Structural and mechanical property predictions of the proposed modelling approach are shown to be in good agreement with the available experimental data. The presented approach is free of parameter-fitting and is capable of generating dispersed Voronoi structures.

## Introduction

Nanoporous open-cellular solids, such as aerogels, demonstrate exceptional properties like ultralow bulk densities and thermal conductivities, as well as very high surface areas. Today, any low-density, sol–gel-derived, predominantly mesoporous (pore-width between 2 and 50 nm) material is widely considered as aerogel. Aerogels can be synthesised from a variety of organic and inorganic sources^1^. While silica aerogels are the most extensively studied ones^1,2^, Kistler described the synthesis of biopolymer aerogels from cellulose, agar, gelatine, and albumin already in 1931^3^. The past two decades have seen an exponential rise in the publications on biopolymer aerogels^4^. This could primarily be attributed to their significant potential in classical aerogel applications such as thermal insulation, as well as emerging applications in filtration, oil–water separation, CO_2_ capture, catalysis, and medicine^5–7^. Aerogels of biopolymers, specifically those from polysaccharides^8–12^, exhibit remarkable mechanical properties that are dependent on their density. These types of aerogels show a fibrillar morphology, where the aerogel network is made up of three-dimensionally interconnecting fibres. This gives them a foam-like appearance on a microstructural level. Many mechanical characteristics, e.g., Young’s modulus $E$ of foam-like open-porous materials maximise with their density $\rho$. This is mathematically interpreted by means of the scaling relationship $E\propto {\rho }^{\alpha }$, where $\alpha$ denotes the scaling exponent. According to the open-cell foam theory by Gibson and Ashby^13^, $\alpha =2$ for a perfectly connected open-cellular solid. For aerogels, it generally lies between 2 and 4^14^. In particular, $\alpha \approx 2$ for cellulose aerogels^7^.

Modifying the pore-structure (pore-volume, pore-area) in such aerogels using sacrificial template methods, results in scaffold-like microstructures^15^. The growing life expectancy and the high obesity ratios of the population lead to more and more frequent bone diseases and traumatic fractures. The inadequacy of donor grafts have strongly motivated the recent advancement in developing synthetic grafts (scaffolds)^16^. An ideal scaffold for tissue engineering purposes must fulfil certain requirements to achieve the desired biological response. Amongst others, the scaffold should exhibit an inter-connected pore structure with appropriate pore-sizes that favour tissue integration and vascularization. It must be synthesised from materials with controlled biodegradability, have appropriate surface chemistry to favour cellular growth, possess reasonable mechanical properties to match the intended site of implantation and handling, and be easily fabricated into a variety of shapes and sizes^17^. In the past 5 years, there have been diverse attempts to address tissue engineering and regenerative medicine problems using polysaccharide-based aerogels as scaffolds^18–21^. Several studies have also explored the in vitro^19,22,23^ and in vivo^21,24^ biological performance of such materials. For optimizing aerogels for tissue engineering and regenerative medicine applications, an in-depth understanding of their pore-structure and their mechanical properties is indispensable.

The current research on biopolymer aerogels is driven forward by experimental developments towards materials discovery at the laboratory scale but lacks a concrete theoretical basis^7^. The mechanical properties of biopolymer aerogels are experimentally tailored by varying the synthesis parameters in a way that it changes their density at the end of the synthesis process. This is subsequently dependent on several factors, e.g., the type of polysaccharide, the solvent, the concentration (wt% or vol%) of the polysaccharide, to name a few. Recently, there have been a few modelling and simulation studies attempting to close the gap between theory and experiments to investigate the structure–property relationships in biopolymer aerogels. Rege et al.^25^ first proposed a micromechanically-motivated constitutive model based on the pore-wall kinematics to describe the macroscopic stress–strain behaviour of cellulose aerogels. Physically motivated model parameters were correlated to experimental ones and the model showed good agreement with the test data. This model was then extended to other biopolymer aerogels, like those from pectin and κ-carrageenan^26^.

Mechanical properties of aerogels are highly influenced by their morphological parameters, such as, the pore-size distribution and the properties of their pore-walls^27^. The pore-size distributions in aerogels are generally evaluated from the output of the nitrogen sorption tests, where the relevant data from the desorption isotherms is exported into the Barrett–Joyner–Halenda (BJH) model^28^. The latter one then provides the pore-area and pore-volume distributions. The above-mentioned material models^25–27^ were among the first to consider the pore-size distribution data as an input parameter for modelling the behaviour of aerogels. While the models accounted for the variation in sizes of square-shaped pores, the random pore shape of aerogels was however not considered. In another approach, Dosta et al.^29^ presented a model of biopolymer aerogels based on the discrete finite element method together with the bonded particle method. They represented the aerogel network at the mesoscale as a set of solid particles connected by solid bonds. An elasto-plastic functional model was developed to describe the mechanical behaviour of alginate aerogels. The model was however not able to capture the fibrillar morphology of the aerogels. In our opinion, such an approach would be better suited for particle-aggregated aerogels.

The primary challenge in modelling aerogels is the description of their highly irregular pore-structure. While techniques such as nanoholotomography have been applied to characterize and reconstruct the nanostructure of other organic aerogels^30^, it is notoriously difficult to be applied to biopolymer aerogels, especially given their pore sizes below 100 nm. Scanning electron microscopy (SEM) also requires the biopolymer aerogel network to be sputtered with gold or platinum. This results in a tailored representation of the actual pore-structure. The inability to reconstruct the three-dimensional (3-d) pore structure represents a bottleneck for computer simulations of biopolymer aerogels for biomechanical applications, such as for example in tissue engineering and regenerative medicine. Recently, a computational model based on two-dimensional (2-d) Voronoi tessellations was proposed to describe biopolymer aerogel network structures^31^. To this end, 2-d Voronoi tessellation-based simulation boxes adhering to the pore-area distributions of cellulose aerogels, were generated. The 2-d Voronoi tessellations were then imported in a finite element program by modelling and discretizing the pore-walls as beam elements. This ensured that all the necessary deformation modes, such as fibre-stretching and -bending, were accounted for. The model results were qualitatively in agreement with available experimental and other modelling studies. However, no quantitative agreement could be achieved as the out-of-plane connectivity of the network could not be considered within the 2-d framework.

In order to overcome the above-mentioned challenges in reconstructing 3-d biopolymer aerogel network structures, a computational model representing the realistic 3-d pore morphologies of biopolymer aerogels using the Laguerre–Voronoi tessellation (LVT) based on random closed packing of polydisperse spheres (RCPPS) was developed and is presented in this paper. As an example, κ-carrageenan aerogels were modelled and simulated. The spatial configuration of the spheres in RCPPS was then used to construct the Voronoi diagram in the Laguerre geometry. The 3-d computationally designed aerogel microstructures are then subjected to compressive deformation to investigate their mechanical properties.

## Results

### Experimental evaluation of κ-carrageenan aerogels

**Figure 1:**
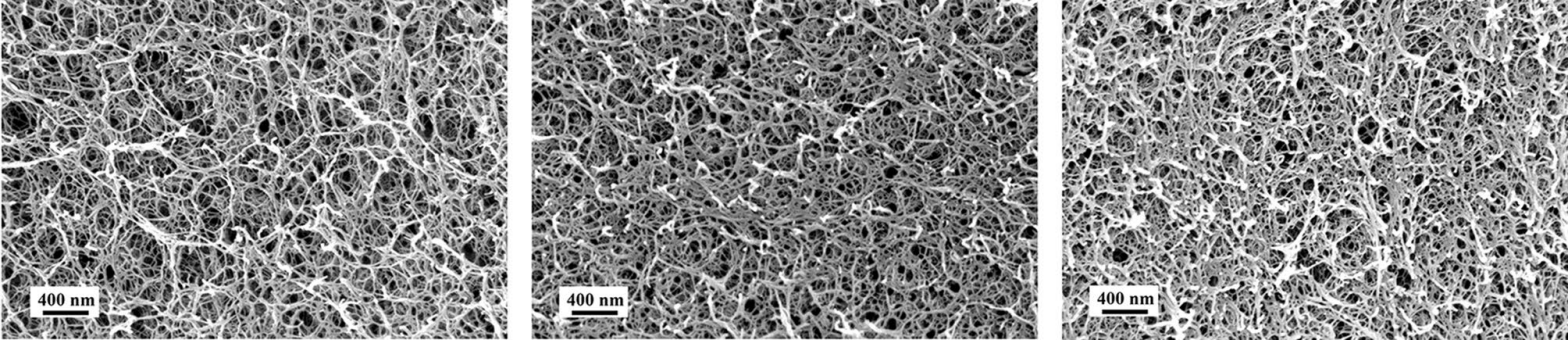
Scanning electron microscopy images of κ-carrageenan aerogels of 1, 2 and 3 wt% (left to right) concentration.

The hydrogels of κ-carrageenan were prepared by addition of potassium thiocyanate which induced the strong gel formation. 1, 2 and 3 wt% concentrations of κ-carrageenan gels were prepared. The hydrogels were strong and rigid. The volume of fresh hydrogel samples measured after the gelation period (16–18 h) was considered as their original volume (${V}_{original}$). The transformation of hydrogels to aerogels by washing, removing potassium thiocyanate, solvent exchange and supercritical drying induced volume shrinkage. Using the volume of the final aerogel as the final volume (${V}_{final}$) in Eq. (1), the total volume shrinkage was calculated and is listed in Table 1. The variation of concentration from 1 to 3 wt% reduced the total volume shrinkage to only 4.5%. On an average, the total volume shrinkage of κ-carrageenan aerogels was about 66%, considering that the small increment in volume shrinkage (about 2.3%) by adding every 1 wt% of κ-carrageenan was steady. This huge volume shrinkage was mainly influenced by the ionic strength of hydrated sulphate functional groups. The morphological properties of the synthesized aerogels are displayed in Table 1. More details can be found in the Supplementary Information. SEM images of κ-carrageenan aerogels are shown in Fig. 1. The fractured surface of the aerogel exhibited the interconnected nano-felt fibre structure. A broad range of pore sizes was observed ranging from meso- to macropores. Increasing the concentration of κ-carrageenan from 1 to 3 wt% also increased the number of interconnected fibres per unit volume. Brunauer–Emmett–Teller (BET) measurements estimate an average fibre diameter to around 10 nm.

**Table 1:** Morphological characteristics of the κ-carrageenan aerogels.

Concentration (wt%)	Volume shrinkage (%)	BET specific surface area (m^2^ g^−1^)	Envelope density (g cm^−3^)	Pore volume (cm^3^ g^−1^)	Porosity (%)
1	68.2 ± 1.6	221 ± 4	0.0714 ± 0.056	1.371	95.9
2	66.2 ± 0.5	228 ± 7	0.1030 ± 0.014	1.974	94.0
3	63.7 ± 0.6	226 ± 6	0.1495 ± 0.0074	1.794	91.3

### Designing a sphere-packed model box

**Figure 2:**
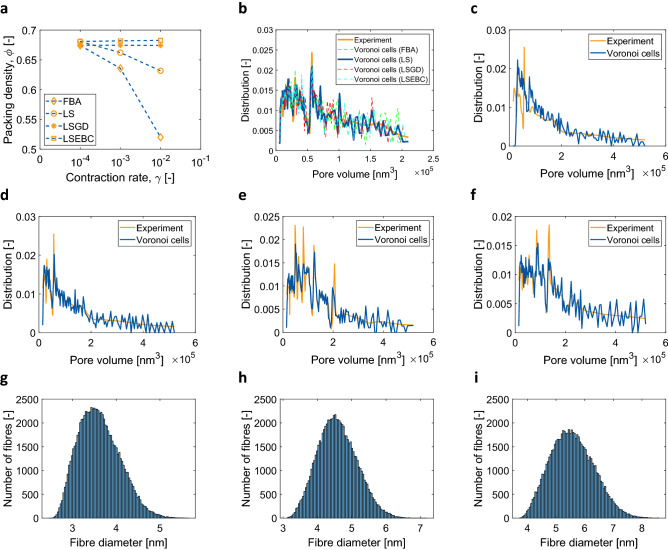
(**a**) Comparison of the packing density of the four RCPPS algorithms FBA, LS, LSGD, and LSEBC, (**b**) PSDs as obtained from the four RCPPS algorithms versus the experimental values for 1 wt% κ-carrageenan aerogel, (**c**) shift arising from the LVT generation over the packed spheres, (**d**–**f**) the LVT-based model vs. the experimental PSD, and (**g**–**i)** the variation in the model fibre diameters for 1, 2 and 3 wt% κ-carrageenan aerogels.

**Figure 3:**
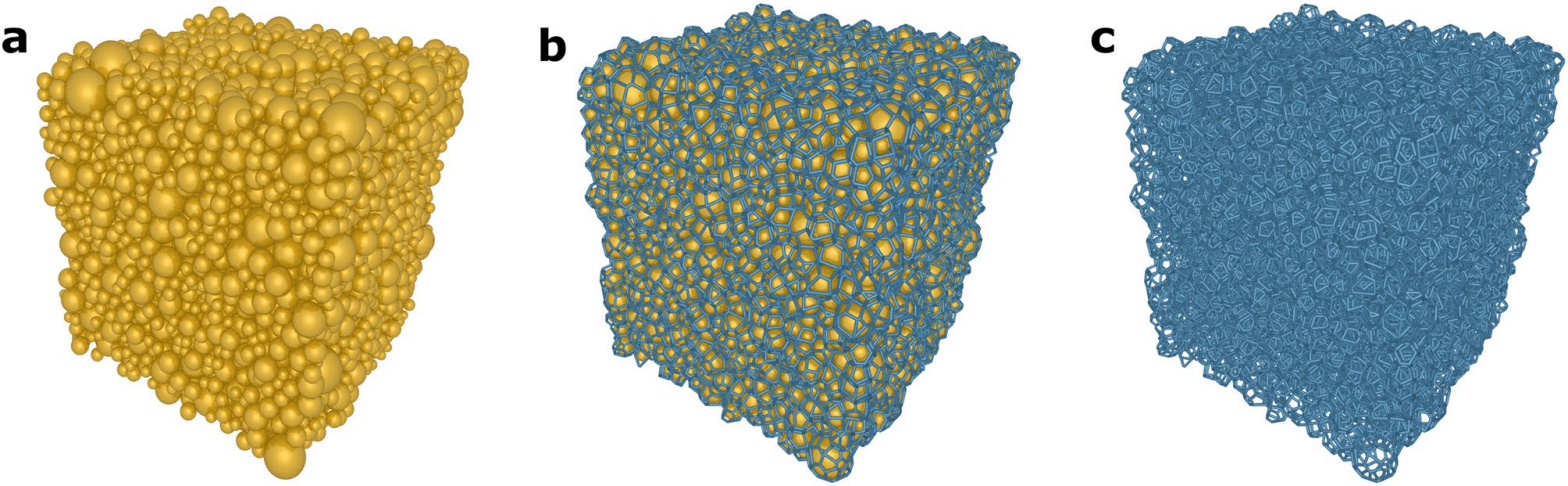
(**a**) Sphere-packed simulation box, (**b**) the corresponding LVT diagram over the sphere-packed box, and (**c**) the computationally designed nanostructured aerogel network corresponding to 1 wt% κ-carrageenan aerogels.

First, the RCPPS was implemented to generate a sphere packing model where the volume distributions of the spheres adhered to those from the pore-size distributions (PSD) of κ-carrageenan aerogels. To this end, four different sphere-packing algorithms were used, namely force-biased algorithm (FBA)^32,33^, Lubachevsky–Stllinger (LS) algorithm^34^, Lubachevsky–Stillinger algorithm with gradual densification (LSGD)^35^, and Lubachevsky–Stillinger algorithm with equilibrium between compressions (LSEBC)^36^. As illustrated in Fig. 2a, dense packing (φ = 0.68) was obtained using LS and LSEBC algorithms with a lower contraction rate (γ = 10^−4^). In Fig. 2b the PSDs as obtained from all the four algorithms are compared *vs*. the corresponding experimentally obtained values. All the above-mentioned algorithms provided good agreement against the experimental data, and thus could be used. However, the LS algorithm was computationally ten times faster in comparison to LSEBC and was thus used for further investigations. Figure 3a exemplarily shows the sphere-packed simulation box for the PSD corresponding to 1 wt% κ-carrageenan aerogel. Images of the simulation boxes corresponding to 2 and 3 wt% κ-carrageenan aerogels are provided in the Supplementary Information.

### Laguerre–Voronoi tessellation

The RCPPS served as an input template for LVT in the generation of the aerogel microstructure, where each Voronoi cell enclosed a sphere and the LVT inherited the volume distribution of the spheres. However, the volume of the Voronoi cells was larger than that of the corresponding spheres due to the space filling of the Voronoi diagram over the packed spheres (see Fig. 2c). As a consequence, a corrector step was introduced, where the volume of the Voronoi cells was gradually shifted by a factor with respect to the sphere volumes. A scaling factor, as a measure between the sphere volume and the corresponding cell volume, was calculated to study the space occupancy of each sphere in the Voronoi cell after LVT. The scaling factor appeared to be below 0.5 for smaller cell volumes and close to 1.0 for larger cell volumes (see Fig. S1 in Supplementary Information), meaning that for the scaling factor of 0.5 and 1.0, 50% and 100% of the cell volumes were occupied by spheres, respectively. A new distribution of the radii was then realised by scaling the radii of the spheres using the evaluated scaling factor. On the basis of these factors, the RCPPS and LVT was carried out to generate the new Voronoi diagram. As a result, the Voronoi diagram strongly inherited the experimental PSD of the aerogels (see Fig. 2d–f). Figures 3b,c exemplarily show the LVT over the sphere-packed simulation box from Fig. 3a and the computationally generated 3-d nanostructured network for 1 wt% κ-carrageenan aerogel, respectively. The corresponding images of 2 and 3 wt% κ-carrageenan aerogels are provided in the Supplementary Information.

### Simulation of the mechanical properties

**Figure 4:**
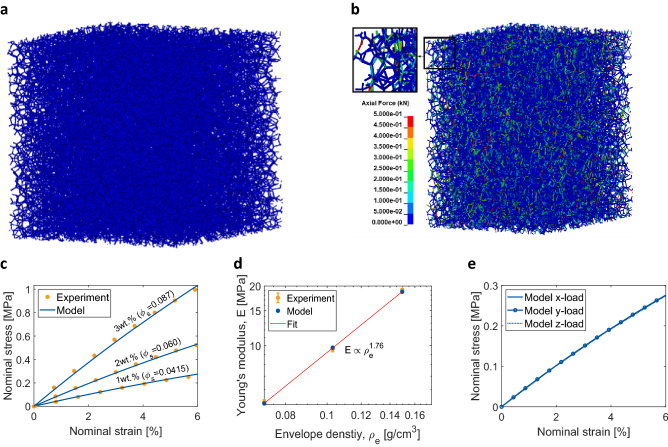
(**a**, **b**) RVEs of 1 wt% κ-carrageenan aerogels in the reference and deformed configuration, (**c**) simulation versus experimental stress–strain curves corresponding to 1, 2 and 3 wt% κ-carrageenan aerogels (${\phi }_{s}$ represents the solid fraction), (**d**) log–log plot of E versus $\rho$ based on the curves in (**c**) showing the value of the scaling exponent $\alpha =1.76$, and (**e**) directional response of the aerogel model under compression in the mutually perpendicular directions x, y and z.

The modelled aerogels were subjected to uniaxial compression in the finite element package LS-DYNA to investigate the macroscopic mechanical behaviour. Periodic boundary conditions were applied for describing the bulk behaviour of the aerogels. Figures 4a,b exemplarily show the representative volume element (RVE) of 1 wt% κ-carrageenan aerogel in the reference and deformed configuration, respectively. Young’s modulus of 4.5 GPa was assigned to the beam elements^37^. While experimental characterisation only reveals an estimation of an average fibre diameter (see “Methods” section), in this work, the fibre diameters were evaluated theoretically from the experimentally obtained PSD and the solid fraction, preserving the porosity of all the three κ-carrageenan aerogels (for more details, see “Methods” section and Fig. S4). The so obtained fibre diameter distributions are shown in Fig. 2g–i. The simulated stress–strain curves are illustrated in Fig. 4c and show good agreement to the corresponding experimental data. Furthermore, the scaling relationship between $E$ and $\rho$ was characterised and is displayed in Fig. 4d. Accordingly, a scaling exponent $\alpha =1.76$, which is close to 2 as given by the open-cell foam model, was obtained. This was also in agreement with the experimentally obtained value of 1.81 ± 0.05. The aerogel RVE was then subjected to uniaxial compressions, separately, along all the three mutually orthogonal directions, to investigate mechanical anisotropy. The respective three stress–strain curves coalesced with each other and are shown in Fig. 4e.

## Discussion

In this study, RCPPS together with LVT was shown to be a powerful method to computationally generate and model the 3-d nanostructured network of aerogels. Dispersed Voronoi structures could be generated. Moreover, the proposed model approach is free of parameter-fitting. Up to date, the approach has been developed and shown to be applicable for fibrillar aerogels, such as those from biopolymers. However, the modelling concept is not limited, and can be optimized for other nano-, micro- and macro-porous open-cellular materials. To demonstrate its applicability to aerogels, κ-carrageenan aerogels were chosen. They represent good examples of fibrillar aerogels from a polysaccharide source. As visualized in Figs. 2d–f and 3c, the developed microstructures adhere strongly to the PSDs of real aerogels from experiments. While all the investigated RCPPS algorithms were able to capture the experimental PSD, the LS algorithm proved to be the most efficient. After the implementation of the LVT-diagram in the sphere-packed simulation box, the obtained open-cellular network structure visually represented the random pore-shapes which can be seen in the SEM micrographs (see Fig. 1). Thus, not only the variation in pore sizes, but also the random pore shapes are accounted for by this model. This is a significant step over the state-of-the-art available modelling tools to describe the nanostructure of aerogels.

The simulated mechanical behaviour of the aerogel RVEs was in good agreement with the experimental data. While this is remarkable, given that the pore-size distribution, the solid fraction and the properties of the fibres are the only parameters of the model, the difference in the calculated fibre diameters and the experimentally estimated average values needs further investigations. In the best case, for 3 wt% κ-carrageenan aerogels, the mean calculated fibre diameter was nearly 6 nm (see Fig. 2i) against the experimentally obtained average value of nearly 10 nm. A possible explanation to this small discrepancy is that the BJH pore-size data accounts for pore-widths only up to ca. 120 nm. SEM images show that there are a few pore sizes even of the order of 400 nm, which cannot be estimated by nitrogen sorption analysis. Accounting for these larger pores, having a comparable pore-wall width as the smaller pores, may result in a lower solid fraction as well as a softer constitutive behaviour. The reverse engineering of the fibre diameter distribution would then yield a distribution with slightly larger mean values, thus shifting towards the experimentally estimated average value. However, without accounting for these larger pore-sizes, the use of a mean value near 10 nm, resulted in a larger solid fraction, thus becoming inconsistent with the material data. In order to avoid losing the physics-informed nature of the model, the calculated diameter distributions were chosen to be the best choice for modelling the aerogels. Since all the input parameters, viz. the pore-size distribution, the solid fraction and Young’s modulus of the fibres, were obtained from analytical tools used to characterise aerogels, the need to fit the model curves was fully eliminated. This is further an important step in modelling the aerogels compared to traditional constitutive modelling, which at least to a moderate extent involves parameter fitting. Moreover, the results also show the importance of pore-sizes and their distributions in the determination of the macroscopic mechanical behaviour of aerogels. While the (relative) density is an important characteristic of porous materials, the significance of pore-size distributions is often underestimated. This appears especially evident for nanoporous materials like aerogels, which show highly varying pore-sizes ranging from micro- to meso- to macro-pores. While the evaluated exponent from the $E$ versus $\rho$ relation is in agreement with the experimental data, it is also very close to that for a perfectly connected open-cellular network. While most of the monolithic aerogel specimens prepared for mechanical testing are cylindrical in shape, characterizing the directional dependency of their mechanical properties is challenging. Thus, this dependency was computationally investigated and the simulated RVEs showed an isotropic behaviour, as the stress–strain responses in x, y and z directions were found to be identical.

This study demonstrates a first-of-its-kind computational reconstruction of the inter-connected pore-structure of biopolymer aerogels, which is highly desirable for computer simulations of scaffolds. The integration of the reconstructed pore-structure in finite elements further shows the capability of simulating nano-, micro- and macro-porous materials under mechanical deformation for biomechanical applications. While designing materials for biomedical applications have been a motivation behind this work, there are endless possibilities to modify, extend and apply this model approach for simulating other porous materials, even those from non-biopolymer sources, for a wide spectrum of applications. These include for example thermal insulation, filtering, CO_2_ adsorption, light-weight design of porous materials as components in, e.g., aircraft cabins^38–40^.

## Methods

### Materials

Potassium thiocyanate and κ-carrageenan were purchased from Merck and Sigma-Aldrich, respectively. They were used as received. Deionized water was used for the preparation of hydrogels. Acetone (NORMAPUR) was used for washing and removal of traces of potassium thiocyanate. Supercritical drying was carried out in an autoclave using pure CO_2_, following the procedure reported by Hoepfner et al.^8^.

### Synthesis of κ-carrageenan aerogels

κ-carrageenan aerogels were prepared by following the procedure described by Ganesan and Ratke^10^. An aqueous dispersion of κ-carrageenan (for example: for 1 wt% gel preparation, 175 mL of water and 2 g of κ-carrageenan) was heated to 95 °C in order to get a clear solution. Then, 25 mL of an aqueous solution of potassium thiocyanate (2.4 M) was added dropwise in a way that the gelation did not take place at above 90 °C. Afterwards, the clear solution was stirred for 5 more minutes. Then, the solution was transferred to moulds and kept at room temperature for 16–18 h in order to complete the gelation. The hydrogels were strong and rigid in the presence of potassium thiocyanate. After washing with acetone, doing solvent exchange and confirming the cleanliness of the gels, the acetone containing gels were subjected to the super critical drying process. The obtained aerogels were white in colour. 1, 2 and 3 wt% concentrations of the aerogel were prepared.

### Characterisation techniques

The purity of the gels was confirmed as per the methods reported in our previous study^10^ in which the acetone washings were titrated against an aqueous solution of iron(III) nitrate (7 mM). The absorbance band at 451 nm was controlled by UV–Vis spectroscopy which corresponds to the iron salt of thiocyanate. The absence of this absorbance band indicated the purity of the gels. UV–Vis absorption spectra were recorded using a Perkin Elmer Lambda 19 by means of 1 cm quartz cuvettes. After drying, the aerogel products were characterised by envelope density measurement (Micromeritics—GeoPyc 1360), skeletal density (Micromeritics—Accupyc II 1340; Gas pycnometer—Helium) and scanning electron microscopy (SEM: Merlin—Carl Zeiss Microscope; gold sputtered samples). BET nitrogen adsorption–desorption isotherm analysis was characterised by Micromeritics—Tristar II 3020. Micromeritics’ innovative MicroActive software was used to evaluate the isotherm data and Barrett–Joyner–Halenda (BJH) pore data analyses. Compression tests on κ-carrageenan aerogel monoliths were performed on a universal testing machine from Latzke using samples of cylindrical shape (10 mm diameter and 10 mm height) and a compression rate of 10%/min. 500 N and 100 N force sensors were used for 2/3 wt% and 1 wt% κ-carrageenan aerogels, respectively.

Percentage of volume shrinkage, porosity, and diameter of fibrils were calculated using the following equations and are given in Table 1. Percentage of volume shrinkage (*V*_*sh*_) was calculated, as follows

1 $${V}_{sh}=\left( 1- \frac{{V}_{final}}{{V}_{original}} \right) \times 100\%.$$

Porosity (ε) was calculated from envelope (*ρ*_*e*_) and skeletal density (*ρ*_*s*_) as follows,

2 $$\upvarepsilon =1-\frac{{\rho }_{e}}{{\rho }_{s}}.$$

The obtained specific surface area (*S*_*m*_) and skeletal density (*ρ*_*s*_) were used to estimate the average fibril diameter (*D*) of κ-carrageenan aerogels, as^10^

3 $$D=\frac{4}{({\rho }_{s }.{ S}_{m})}.$$

### Random closed packing of polydisperse spheres (RCPPS)

**Figure 5:**
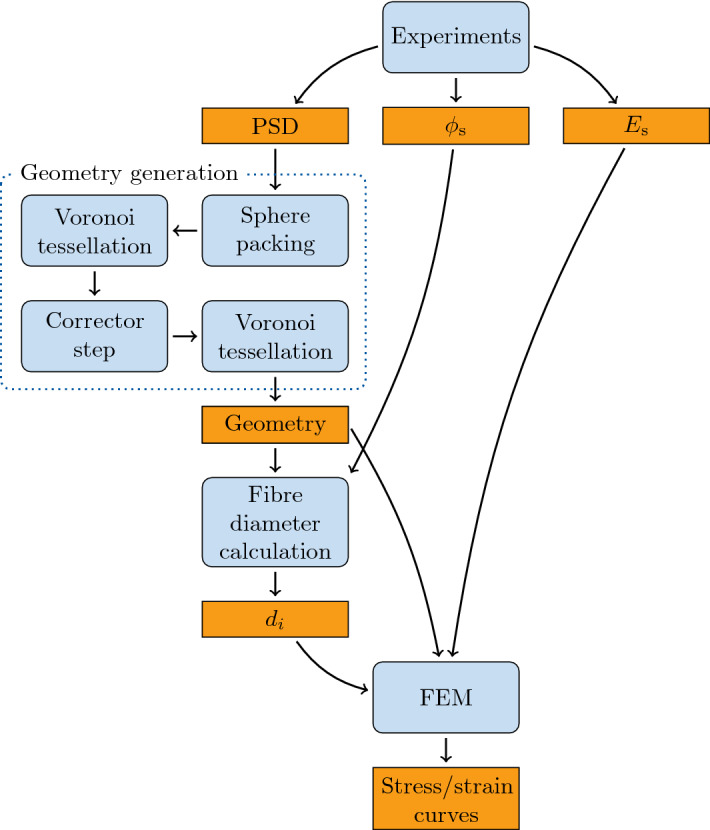
Flowchart demonstrating the flow of the overall modelling process, from experimental data acquisition over the geometry generation and optimisation to the finite element analysis. The orange boxes denote the usable outputs from experiments or the model, while the blue boxes represent the processes involved in obtaining these outputs.

RCPPS can be generated by computer simulation using different algorithms based on sequential generation/sedimentation technique and collective rearrangement models^32^. The former technique based on drop and roll method can be further classified into two types: (1) vertically dropping of the spheres onto the surface of an existing particle cluster which grows upward, and (2) centripetally placing on the surface of a centre cluster which grows outward. The collective rearrangement models generate randomly distributed initial positions of sphere within the packing domain with many overlaps and use a relaxation process to separate the overlapping spheres. The sequential generation/sedimentation technique produces slightly anisotropic microstructures^41^, while the collective rearrangement models produce dense isotropic packings and hence were used in this work^42,43^. In particular, the forced-biased algorithm^33^ (FBA) and the Lubachevsky–Stillinger^32,34^ (LS) algorithm, classified as a collective rearrangement method, were used to generate RCPPS by following Baranau et al.^35,36^ The principle of LS algorithm is based on a molecular dynamics simulation, where the particle radii are gradually increased with a certain expansion rate until the predefined pressure value is reached^34^. A lower expansion rate generates a denser packing. A successful packing is obtained using the LS algorithm only when the initial packing has a relatively large packing density (0.4 to 0.6), because a collision with particles far away from each other is not considered. Therefore, Baranau et al.^35,36^ suggested to obtain an initial packing for LS algorithm using FBA^33^. In FBA, the particles were randomly distributed in space with corresponding inner and outer diameter defined. The algorithm attempts to reduce the overlap between the spheres in every step (a) by pushing apart overlapping spheres with new position and (b) gradually shrinking the spheres by reducing the outer diameter. The proposed process methodology is sketched in Fig. 5. The RCPPS only uses the pore-size distribution from experiments and generates the sphere packing as described above and in the Results section.

### Laguerre–Voronoi Tessellation (LVT)

A tessellation is a finite division of $m$-dimensional space into different polytopes connected to each other, which can be used to generate topological models. The most well-known classical method is the Voronoi tessellation^44^. The Voronoi diagram is constructed by convex polygonal cells with a set **S** = {${s}_{1},{s}_{2},\dots , {s}_{n}$} of $n$ randomly distributed seed points in ${\mathbb{R}}^{m}$. The cell edges are generated equidistant to the two closest seed points in **S**. A Voronoi cell corresponding to seed point ${s}_{i}$ is defined as

4 $$V\left( {s_{i} } \right) = \left\{ {x \in {\mathbb{R}}^{m} |\left\| {x - s_{i} } \right\|_{2} \le \left\| {x - s_{j} } \right\|_{2} } \right\} \;\;\;\forall j = 1,2, \ldots ,n :j \ne i,$$

where $x$ represents the position of any point in the $m$-dimensional space and the normal Euclidean distance between ${s}_{i}$ and any point $x$ is denoted by $\left\| \cdot \right\|_{2}$. Due to the equidistant generator property, the range of cell patterns realised by Voronoi tessellations is limited, i.e., it is unable to model a highly polydisperse microstructure. Additionally, the Voronoi tessellation constructs cells based on randomised seed point coordinates. This makes it very difficult to inherit the PSDs of aerogels. As a consequence, Laguerre–Voronoi tessellation (LVT), also referred to as the radical or power tessellation, was evaluated in this work. LVT is a weighted version of the Voronoi tessellation, where each point ${s}_{i}$ is assigned with a weight ${\omega }_{i}$. The relative value of the weights ${\omega }_{i}$ of two neighboring seeds determines the position of the cell boundaries. Accordingly, $V({s}_{i})$ in Eq. (4) is modified by ${\Vert x-{s}_{i}\Vert }_{2}-{\omega }_{i} \le {\Vert x-{s}_{j}\Vert }_{2}-{\omega }_{j}.$ In LVT, each Voronoi cell is generated based on a sphere with a specified radius and centre coordinate^45,46^. As a result, the cell boundaries can be generated as tangent to the sphere surface at the point of contact between two adjacent spheres of different sizes rather than equidistance positioning. The RCPPS provides the seed point set (**S**) in ${\mathbb{R}}^{m}$, consisting of coordinates of all sphere centres and the weight set ${\varvec{\upomega}}$ = {${r}_{1}^{2},{r}_{2}^{2},\dots , {r}_{n}^{2}$}, calculated from the corresponding sphere radii. A Voronoi cell generated using LVT for each sphere with seed point ${s}_{i}$ is defined as

5 $$V_{L} \left( {s_{i} } \right) = \left\{ {x \in {\mathbb{R}}^{m} |D_{L} \left( {x,s_{i} } \right) \le D_{L} \left( {x,s_{j} } \right)} \right\} \quad \forall j = 1,2, \ldots ,n :j \ne i,$$

where ${D}_{L}\left(x,{s}_{i}\right)={\left[{\Vert x-{s}_{i}\Vert }^{2}-{r}_{i}^{2}\right]}^{1/2}$ defines the distance between ${s}_{i}$ and any point $x$. Thus, the sphere packing is used as a template to construct the Voronoi cells as described above and also illustrated in Fig. 5. There is a corrector step involved wherein the pore-size distribution of the Voronoi structure is scaled to the original experimental pore-size distribution (as discussed earlier in the Results section). Thus, an optimised Voronoi-based structure adhering to the experimental pore-size distribution is generated.

### Determination of the fibre diameter

Once the Voronoi-based fibrillar microstructure is computationally generated, a fibre diameter needs to be assigned to every fibril. Experimental characterisation estimates an average fibre diameter, either from BET measurements or from SEM image evaluation, for the entire meso-macro-porous aerogel network. In this work, the fibre diameters were calculated theoretically from the solid fraction of the aerogels by preserving its porosity as experimentally determined (Table 1). The volume of all the fibres constituting one pore was calculated from the total volume of the pore and the porosity. The volume share of each pore-wall of a given pore was then quantified using their lengths. Each fibre is shared by more than one pore, and consequently the volume share of the fibres in each pore was added up to determine the total volume of the fibres. Subsequently, the fibre diameters were then calculated from their total volumes and lengths. While the flowchart in Fig. 5 roughly illustrates the processes involved in calculating the fibre diameters, a more detailed step-by-step algorithm is presented in Fig. S4.

### Finite element modelling

The Voronoi diagram generated using LVT based on RCPPS is transformed to a cube shaped RVE with periodic boundary nodes^47^. The cell edges of the Voronoi structure represent the cell wall fibres in the aerogel network, which are imported as line bodies and meshed as beam elements with a circular cross-section in LS-DYNA. These are assigned diameters as described earlier. The Hughes–Liu beam element formulation with cross-section integration is used. It is based on the degenerated brick element formulation incorporating finite transverse shear strains with bending and membrane capabilities. The beams were meshed only for fibres larger than the diameter of their cross-section. A mesh size of 5 nm was used, although it did not influence the macroscopic response of the 3-d model under compression. An automatic general contact algorithm is used to model the beam-to-beam contact, in order to avoid the penetration between the fibre walls. An RVE in combination with periodic boundary conditions (PBCs) on its boundaries has the potential to obtain the homogenized macroscopic response. Using PBCs, the motion of boundary node pairs is constrained to each other, such that the stress continuity across the boundaries is preserved during deformation.

## Supplementary Information

Supplementary Information.
