# Supplementary material for: Computational design of biopolymer aerogels and predictive modelling of their nanostructure and mechanical behaviour
Source: Sci Rep. 2021 May 13;11:10198. doi: 10.1038/s41598-021-89634-1 (PMC8119483; doi:10.1038/s41598-021-89634-1)
Supplement: Supplementary file 1 — Supplementary Information. [file 41598_2021_89634_MOESM1_ESM.pdf]

Supplementary Information

Computational design of biopolymer aerogels and predictive modelling of their nanostructure and mechanical behaviour

Rajesh Chandrasekaran[1, *], Markus Hillgärtner[1], Kathirvel Ganesan[2], Barbara Milow[2], Mikhail Itskov[1] and Ameya Rege[2, *]

[1]Department of Continuum Mechanics, RWTH Aachen University, Eilfschornsteinstr. 18, 52062 Aachen, Germany

[2]Department of Aerogels and Aerogel Composites, Institute of Materials Research, German Aerospace Center, Linder Höhe, 51147 Cologne, Germany

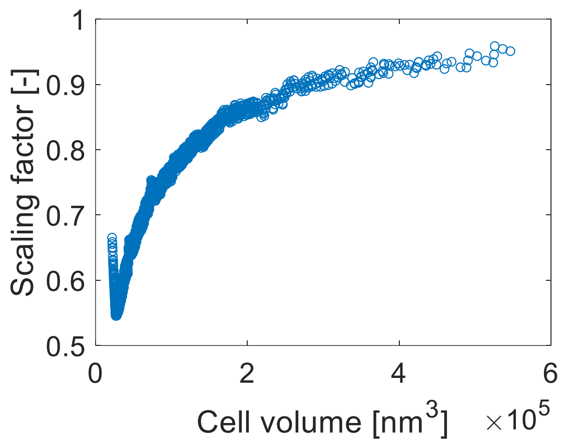

Figure S1: Scaling factor to obtain the Voronoi cell volume distribution

Additional details about Table 1. The envelope density increased from 0.07 to 0.15 g cm^{-3} with the increase of κ-carrageenan concentration from 1 to 3 wt.%, respectively. Brunauer-Emmett-Teller (BET) specific surface area was in the range between 221-228 m^2 g^{-1} showing no significant influence while increasing the concentration from 1 to 3 wt.%. Recently, this synthetic procedure was reproduced by Agostinho *et al*. (Materials Chemistry and Physics 253: 123290 (2020)), and the data of specific surface area and total volume shrinkage was in good agreement with our previous work (Ganesan and Ratke, Soft Matter 10:3218 (2014)). The mesopore volume of aerogels was in the range between 1.37 and 1.97 cm^3 g^{-1}. The lower the concentration of κ-carrageenan, the lower was the porosity of the aerogels. The skeletal density (ρ_s) of κ-carrageenan was 1.72 g cm^{-3} as obtained from helium pycnometer. Using this value in Eq. (3), the fibre thickness was estimated, and it was in the range between 10.2 and 10.55 nm. It indicated that the fibre thickness was not significantly influenced by increasing the concentration of κ-carrageenan.

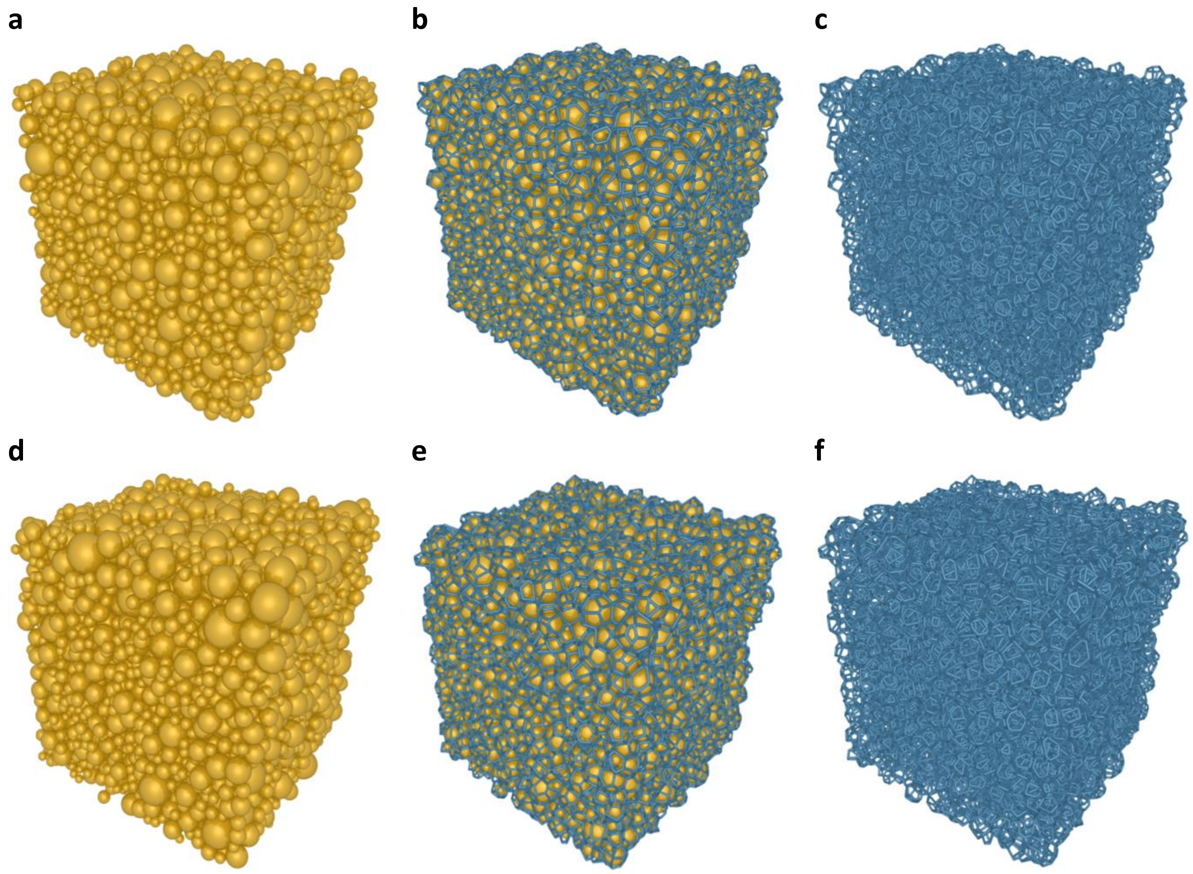

Figure S2**: a, d** Sphere-packed simulation box, **b, e** the corresponding LVT diagram over the sphere-packed box and **c, e** the computationally designed nanostructured aerogel network corresponding to 2 wt.% and 3 wt.% κ-carrageenan aerogels, respectively.

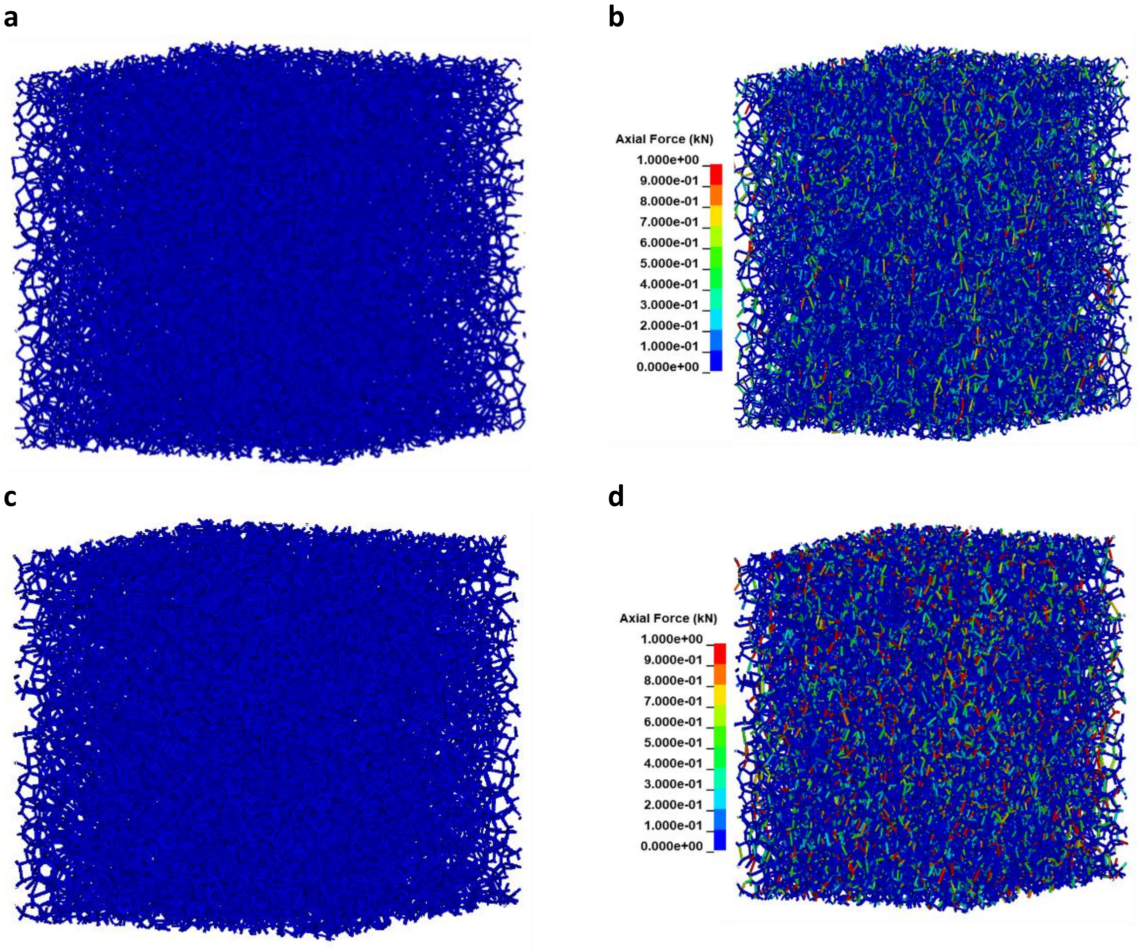

Figure S3: The RVE in the reference and deformed configuration of **a-b** 2 wt.% and **c-d** 3 wt.% κ-carrageenan aerogels, respectively.

Algorithm 1 Calculate fibre diameter

Calculate the volume of each Voronoi cell: V_j
Calculate the solid fraction of each Voronoi cell: $\bar{V}_j = \phi_s V_j$
Initialise the edge volume to 0 for all edges

for each Voronoi cell **do**
    Calculate the set of edges adjacent to the cell: S
    Calculate the sum of the edge lengths in S: L_S
    Calculate weighting factors for each edge in S based on its length$/L_S$
    Calculate the volume share for each edge in S using $\bar{V}_j$ and the weighting factors
    Add the determined edge volume share to the edge volumes

for each edge **do**
    Calculate the required fibre diameter based on the edge volume

return fibre diameter for each edge

Figure S4: Algorithm illustrating the step-by-step procedure to obtain individual fibre diameters in the computationally generated Voronoi-based aerogel microstructure.
